# Rigid ureteroscopic lithotripsy versus percutaneous nephrolithotomy for large proximal ureteral stones: A meta-analysis

**DOI:** 10.1371/journal.pone.0171478

**Published:** 2017-02-09

**Authors:** Qing Wang, Jiachao Guo, Henglong Hu, Yuchao Lu, Jiaqiao Zhang, Baolong Qin, Yufeng Wang, Zongbiao Zhang, Shaogang Wang

**Affiliations:** 1 Department of Urology, Tongji Hospital, Tongji Medical College, Huazhong University of Science and Technology, Wuhan, Hubei, China; 2 Department of Orthopedics, Tongji Hospital, Tongji Medical College, Huazhong University of Science and Technology, Wuhan, Hubei, China; Peking Union Medical College Hospital, CHINA

## Abstract

**Object:**

To compare the safety and efficacy of rigid ureteroscopic lithotripsy (rigid URSL) and percutaneous nephrolithotomy (PCNL) in treating large proximal ureteral stones.

**Methods:**

A systematic search of PubMed, EMBASE, Cochrane Library, and Web of Science databases was performed to find out relevant studies. After literature screening according to the predetermined inclusion and exclusion criteria, data of eligible studies was extracted and then a meta-analysis was conducted via RevMan 5.3 software.

**Results:**

Five randomized controlled trials (RCTs), one prospective and four retrospective cohort studies involving 837 patients were included. Patients underwent rigid URSL were associated with shorter operation time (WMD, -23.66min; 95%CI, −45.00 to -2.32; p = 0.03), shorter hospital stay (WMD, -2.76d; 95%CI, −3.51 to −2.02; p< 0.00001), lower 3rd-day (RR, 0.73; 95%CI, 0.66 to 0.82; p < 0.00001) and 1st-month (RR, 0.82; 95%CI, 0.77 to 0.87; p < 0.00001) stone-free rate, higher risk of conversion to other surgical procedures (RR, 4.28; 95%CI, 1.93 to 9.46; p = 0.0003), higher incidence of migration (RR, 28.49; 95%CI, 9.12 to 89.00; p < 0.00001) and ureteral perforation (RR, 6.06; 95%CI, 1.80 to 20.44; p = 0.004), lower risk of fever (RR, 0.64; 95%CI, 0.42 to 0.97; p = 0.04), transfusion (RR, 0.19; 95%CI, 0.04 to 0.85; p = 0.03) and hematuria (RR, 0.38; 95%CI, 0.25 to 0.57; p < 0.0001). No significant difference was observed in terms of incidence of embolization, pain and ureterostenosis. When cohort studies or studies in which flexible ureteroscopy was used as an intraoperative auxiliary procedure were excluded, we both found that most of the results kept stable.

**Conclusions:**

Both PCNL and rigid URSL are safe for patients with large proximal ureteral stones while PCNL is more effective in stone clearance.

## Introduction

Large proximal ureteral stones can lead to urinary obstruction, which may be followed with renal function injury and life-threatening sepsis. Timely intervention to remove the stones completely is of great importance while the most appropriate treatment remains controversial.

According to the latest American Urological Association (AUA) Guideline on surgical management of stones, extracorporeal shock wave lithotripsy (SWL) and ureteroscopic lithotripsy (URSL) have been proposed as the first-line treatments for proximal ureteral stones [1]. However, SWL should not be a priority for stones larger than 10mm because of dramatically decreased stone-free rate and requirement of multiple sessions [2,3]. 2016 European Association of Urology (EAU) Guideline indicates that percutaneous nephrolithotomy (PCNL) can be considered in selected cases, such as large (>10mm), impacted proximal ureteral calculi with dilated renal collecting system, or when the ureter is not amenable to retrograde manipulation [4].

Both URSL and PCNL are widely used as minimally-invasive treatments for large proximal ureteral stones. Rigid URSL is the most used ureteroscopy technique and the reported stone-free rate for it in managing upper ureteral stones ranges from 88% to 100% [5,6]. PCNL also shows a high stone clearance rate in proximal ureteral stones since it was introduced into routine clinical practice in 1980s [7,8,9]. Despite the reliable efficiency, each technique has its own limitations. Migration of stones or fragments is the main reason for failure in rigid URSL and further auxiliary procedures, such as flexible URSL and SWL, are often required in this case. PCNL is a more invasive technique, during which bleeding is generally common and 0–20% with an overall of 7% need transfusion [10]. Moreover, adjacent organ injury should not be ignored when referring to PCNL even though the incidence is only about 0.4% [11]. So there comes a question for urologists that which one is better for patients with large proximal ureteral stones. Since 1999, several studies comparing the efficacy and safety of rigid URSL and PCNL in treating large proximal ureteral stones have been carried out [12–21]. As the results of these studies are not totally consistent, a meta-analysis which compares the outcomes of rigid URSL and PCNL should be carried out to provide some advice for urologists and patients in making relevant decisions in the future.

## Materials and methods

### Literature search

We conducted a systematic literature search of Medline (using PubMed as the search engine), Embase (using Ovid as the search engine), Web of Science databases and the Cochrane Library to identify relevant studies in accordance with PRISMA (Preferred Reporting Items for Systematic Reviews and Meta-Analyses)[22] in April 2016 and updated in August 2016. The search was performed with the following terms: (“ureteroscope” or “ureteroscopy” or “ureteroscopic lithotripsy” or “ureterolithotripsy” or “retrograde”) and (“percutaneous nephrolithotomy” or “percutaneous lithotripsy” or “PCNL” or “PNL” or “PCN” or “antegrade”) and (“ureteral calculi” or “ureteral calculus” or “ureteral stone” or “ureteral stones”). No restriction of year or language was imposed. Two independent reviewers screened all the citations and abstracts. Studies involving comparison of rigid URSL and PNL in treating proximal ureteral stones were included for further screening.

#### Inclusion criteria and exclusion criteria

Studies meeting the predetermined criteria were included: (1) patients with large (>10mm) proximal ureteral stones or accompanied with secondary renal stones (<10mm), (2) comparing rigid URSL with PCNL or including a comparison of rigid URSL and PCNL, (3) both surgical techniques should be performed on adults, (4) the full text could be accessed online, (5) reporting at least one of clinical outcomes of interest (described in data extraction part).

Exclusion criteria included: (1) the study was conducted during pregnancy, (2) conference abstracts (because they seemed not methodologically appropriate), (3) no outcome of interest was reported or it was impossible to calculate, (4) the surgical procedure was performed via specialized technique, for example, PCNL in a modified position, tubeless PCNL, or with the aid of patented systems.

Two reviewers independently completed this selection process. Disagreements were resolved by discussion until a consensus was reached.

#### Study quality and level of evidence

The criteria provided by the Oxford Center for Evidence-Based Medicine [23] was used to rate the level of evidence for all studies. The methodological quality of the non-randomized controlled trials (nRCTs) was assessed using the Newcastle-Ottawa Scale [24] and the Cochrane Collaboration’s tool [25] was used to assess the risk of bias of randomized controlled trials (RCTs). Two reviewers performed the procedure independently. Disagreements were resolved by discussion until a consensus was reached.

### Data extraction

The following data was extracted independently by two reviewers using a pre-designed data extraction form, which consisted of study name, the first author, year of publication, country, study design, number of patients, stone size, age, gender, stone side and clinical outcomes of interest (the 3^rd^-day or the 1^st^-month stone free rate, operation time, hospital stay, and the incidence of intra- or post-operative complications involving stone migration, ureteral perforation, conversion to other surgical procedure, fever, transfusion, embolization, pain, hematuria and ureterostenosis). Type of ureteroscope and nephroscope and whether a postoperative auxiliary SWL was given to patients with residual stones were also extracted in each study, which was critical for the following subgroup and sensitivity analysis.

### Statistical analysis

A meta-analysis was conducted via RevMan 5.3 software of the Cochrane Collaboration to compare the efficacy and safety of rigid URSL with PCNL in treating large proximal ureteral stones. Relative risk or odds ratio was used for dichotomous data, and weighted or standardized mean difference was used for the continuous data. Standard deviations were calculated using the methodology described by Hozoand associates if continuous data was presented as means and range [26]. All the outcomes were reported with 95% confidence intervals (95% CI). The chi-square test and I^2^ value were used to identify the heterogeneity among studies. Pooled estimates were firstly calculated with the fixed-effect model. However, if significant heterogeneity (I^2^ > 50%) was detected and it could not be dissolved by subgroup analysis, the random-effect model was used. The pooled effects were determined by the z test and p < 0.05 was considered statistically significant. Moreover, a sensitivity analysis was performed by pooling only RCTs or studies in which flexible ureteroscopy was not mentioned as an intraoperative auxiliary procedure during rigid URSL.

## Results

### Characteristics of eligible studies

**Fig 1:**
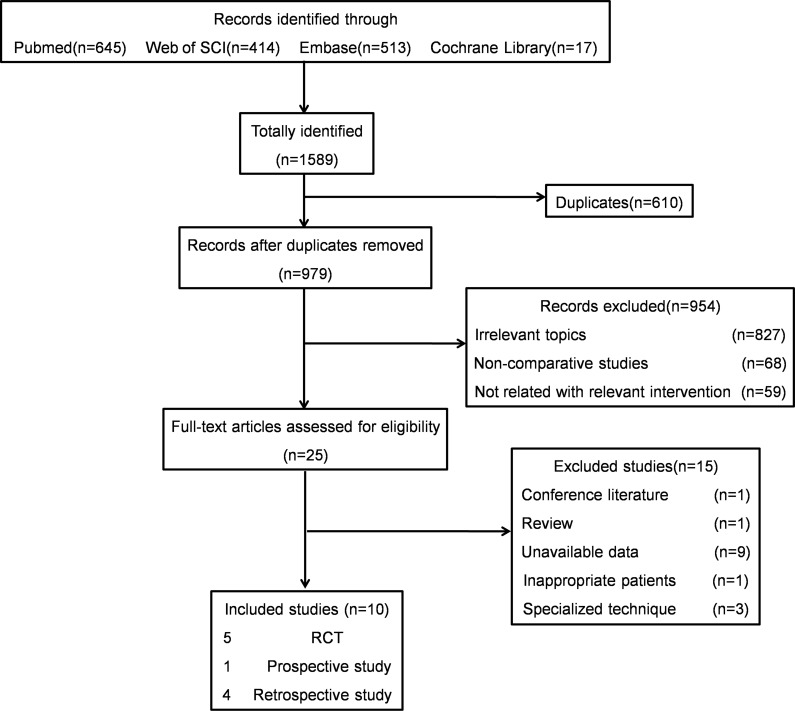
Flowchart of the literature search and studies selection.

Ten studies, involving 417 patients who underwent rigid URSL and 420 underwent PCNL, were included in our meta-analysis. Fig 1 shows the procedure of literature search and study selection. The studies consisted of five RCTs [12–16], one prospective [19] and four retrospective cohort studies [17,18,20,21]. Basic characteristics, such as age, sex ratio, stone size and stone side, were described comparable between rigid URSL and PCNL group in each study and the data was presented in Table 1. Some studies also reported other basic characteristics that might make a difference in the clinical outcomes: preoperative renal function including serum creatinine (SCr), blood urea nitrogen (BUN) or golomeruar filtration rate (GFR), failed SWL history, and degree of hydronephrosis [14,15,17,19,20]. All the data was also comparable between the two groups according to each study.

**Table 1:** Characteristics and methodological quality of included studies.

		Study		Study	Surgical		Sample	Age	Sex		Stone side	Postoperative
Reference, year	Nation	design	LOE	quality^*^	technique	Type of facility	size	(year)	(M/F)	Stone burden	(left/right)	SWL
								(M±SD)				
Sun et al, 2008	China	RCT	2	-	URSL	Rigid ureteroscope	47	39.6±7.3	31/16	14.6±1.8 mm	ND	No
					mini-PCNL	Rigid ureteroscope	44	40.4±8.4	30/14	14.7±2.0 mm	ND	
Basiri et al, 2008	Iran	RCT	2	-	URSL	Semi-rigid ureteroscope	50	39±15	33/17	17.8±2.4 mm	26/22	No
					PCNL	Rigid nephroscope	50	48±13	32/18	20.3±3.3 mm	31/19	
Liu et al, 2013	China	RCT	2	-	URSL	Rigid ureteroscope	45	43.41±10.17	25/20	148.13±27.52mm^3^	23/22	Yes
					mini-PCNL	Rigid ureteroscope	45	46.35±0.31	23/22	146.85±30.36mm^3^	24/21	
Gu et al, 2013	China	RCT	2	-	URSL	Semi-rigid, flexible ureteroscope	29	44.22± 13.0	17/12	16.23(15–25)mm	12/17	Yes
					mini-PCNL	Rigid ureteroscope	30	42.5 ± 10.1	17/13	17.27(15–25)mm	16/14	
Qi et al, 2014	China	RCT	2	-	URSL	Semi-rigid, flexible ureteroscope	52	42.5±10.3	31/21	19.8±4.3mm	30/22	Yes
					PCNL	Rigid nephroscope	52	41.1±12.4	30/22	20.3±3.6mm	27/25	
Maheshwari et al,	India	CS	3	7/9	URSL	Rigid ureteroscope	20	ND	14/6	ND	ND	Yes
1999					PCNL	Rigid ureteroscope,	23	ND	15/8	ND	ND	
Juan et al, 2008	China	CS	3	8/9	URSL	Semi-rigid ureteroscope	31	48.9 ± 12.5	23/8	18.6 ± 6.3mm	18/13	No
					PCNL	Rigid nephroscope, semi-rigid ureteroscope	22	48.2 ± 11.2	16/6	20.1 ± 5.4mm	13/9	
Li et al, 2013	China	CS	3	9/9	URSL	Semi-rigid, flexible ureteroscope	91	45.35±12.51	44/47	20.61±4.26 mm	45/48^**^	No
					PCNL	Rigid nephroscope	83	44.12±11.56	46/37	20.00±4.44mm	39/44	
Zhu et al, 2014	China	CS	3	8/9	URSL	Semi-rigid ureteroscope	22	49.6±7.1	14/8	1.2±0.8cm	12/10	Yes
					PCNL	Rigid nephroscope	30	51.9±8.4	18/12	1.4±0.7cm	17/13	
Bozkurt et al,	Turkey	CS	3	8/9	URSL	Semi-rigid, flexible ureteroscope	41	42.1 ± 14.9	27/18^#^	261 ± 47mm^2^	ND	No
2015					PCNL	Rigid nephroscope	45	44.7 ± 16.3	22/19^#^	314 ± 64mm^2^	ND	

RCT: randomized controlled trial CS: cohort study LOE: level of evidence URSL: ureteroscopic lithotripsy PCNL: percutaneous nephrolithotomy SWL: extracorporeal shock wave lithotripsy ND: not demonstrated.

*The quality of the non-randomized controlled trials (nRCTs) was assessed using the Newcastle-Ottawa Scale.

** Totally 91 patients were included in the URSL group, the authors didn’t mention bilateral stones and they might report a wrong ratio of stone side.

# The author might report the gender ratio inversely in two groups.

### Quality of the studies

**Fig 2:**
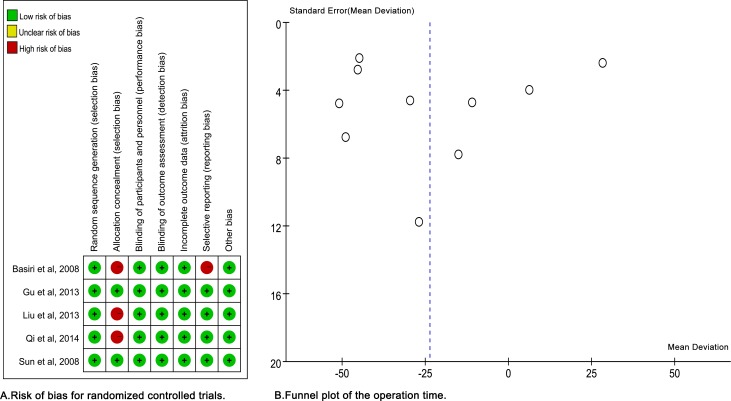
(A) Risk of bias for randomized controlled trials. (B) Funnel plot of the operation time.

As shown in Table 1, all RCTs were rated Level 2 and cohort studies were rated Level 3.Fourcohort studies [17–20] scored ≥8 stars were considered to be of high quality while one study [21] was scored 7 stars. Fig 2A showed that all of the RCTs described suggested randomization. Three studies [12,13,16] failed to report details about allocation concealment. It was quite difficult to perform blinding of participants or personnel in surgical treatment, so a high risk of bias was judged in this part for each study. The blinding outcome measurement was judged to low risk of bias because the outcomes were unlikely to be influenced by lack of blinding. One study [13] had a high risk of selective reporting bias for lack of detailed explanation for some important outcomes. All studies had low risk of other biases. Finally, two RCTs [14,15] were judged to be of high methodological quality while the other three RCTs [12,13,16] were of low quality.

### Publication bias

The publication bias was explored via funnel plots. As showed in Fig 2B, the funnel plot of operation time included all studies and seemed asymmetric, which might be explained by publication bias and heterogeneity.

### Operation time

**Fig 3:**
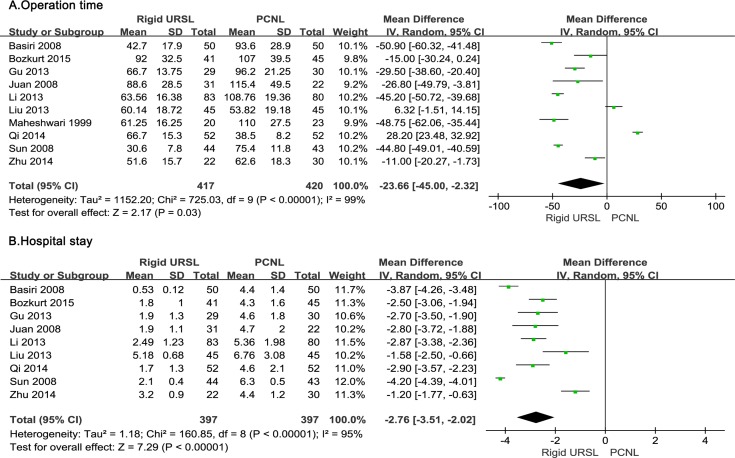
Forest plots of (A) operation time, (B) hospital stay.

Pooling the data from ten studies [12–21] that assessed operation time showed less time in rigid URSL group than in PCNL group(WMD, -23.66min; 95%CI, −45.00 to -2.32; p = 0.03; Fig 3A).

### Hospital stay

Meta-analysis of nine studies [12–20] by a random effects model showed that the rigid URSL group charged with a shorter hospital stay of 2.76d than the PCNL group (WMD, -2.76d; 95%CI, −3.51 to −2.02; p< 0.00001; Fig 3B).

### The 3^rd^-day stone-free rate

**Fig 4:**
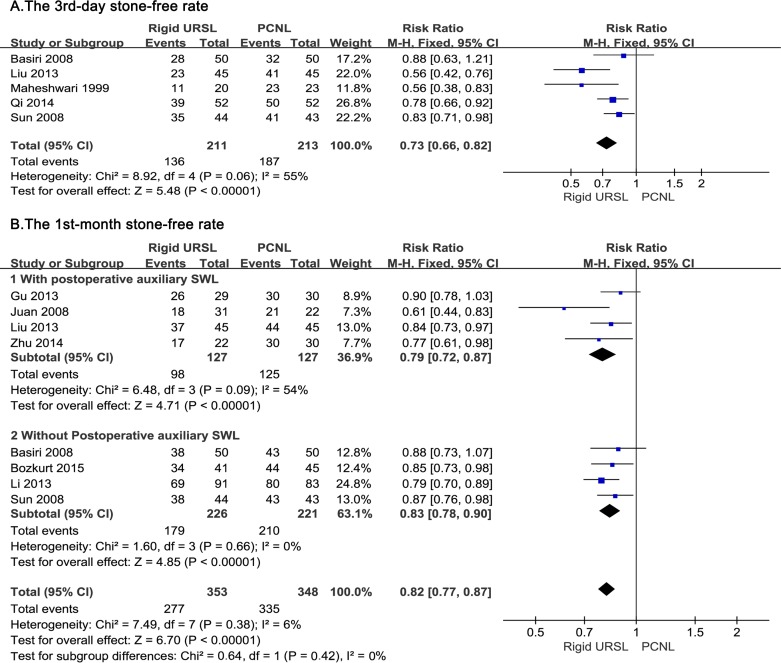
Forest plots of (A) The 3^rd^-day stone-free rate, (B) The 1^st^-month stone-free rate with and without postoperative auxiliary SWL.

Pooling the data from five studies [12,13,15,16,21] demonstrated that the 3^rd^-day stone-free rate of rigid URSL group was significantly lower than that of PCNL group (RR, 0.73; 95%CI, 0.66 to 0.82; p < 0.00001; Fig 4A).

### The 1^st^-month stone-free rate

In terms of the 1^st^-month stone-free rate, the studies were divided into two subgroups according to whether the patients with residual stones received a postoperative auxiliary SWL within the 1st month. Meta-analysis of four studies [14,16,18,20] referring to postoperative auxiliary SWL showed that the 1^st^-month stone-free rate of rigid URSL group was lower than that of PCNL group (RR, 0.79; 95%CI, 0.72 to 0.87; p < 0.00001; Fig 4B). “Without postoperative auxiliary SWL” subgroup consisted of four studies [13,15,17,19] and the analysis also revealed that the 1^st^-month stone-free rate of rigid URSL group was lower than that of PCNL group (RR, 0.83; 95%CI, 0.78 to 0.90; p < 0.00001; Fig 4B). Total effect of the two subgroups indicated lower 1^st^-month stone-free rate in rigid URSL group than in PCNL group (RR, 0.82; 95%CI, 0.77 to 0.87; p < 0.00001; Fig 4B).

### Intraoperative complications

**Fig 5:**
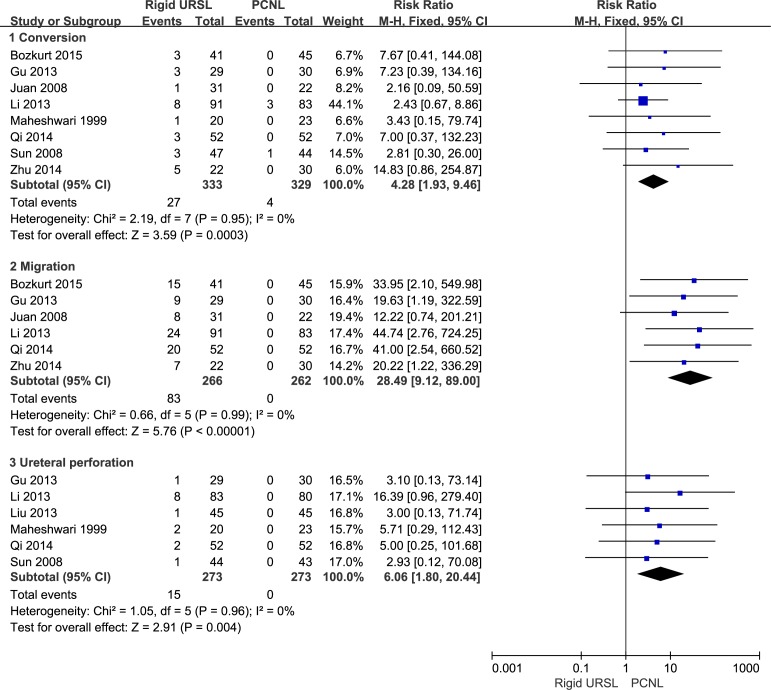
Forest plots of conversion, migration and ureteral perforation.

Patients who underwent rigid URSL, rather than PCNL, were associated with a higher risk of conversion to other surgical procedures (RR, 4.28; 95%CI, 1.93 to 9.46; p = 0.0003; Fig 5) [12,14,15,17–21], migration (RR, 28.49; 95%CI, 9.12 to 89.00; p < 0.00001; Fig 5) [12,14,17–20]and ureteral perforation (RR, 6.06; 95%CI, 1.80 to 20.44; p = 0.004; Fig 5) [12,14–16,19,21].

### Postoperative complications

**Fig 6:**
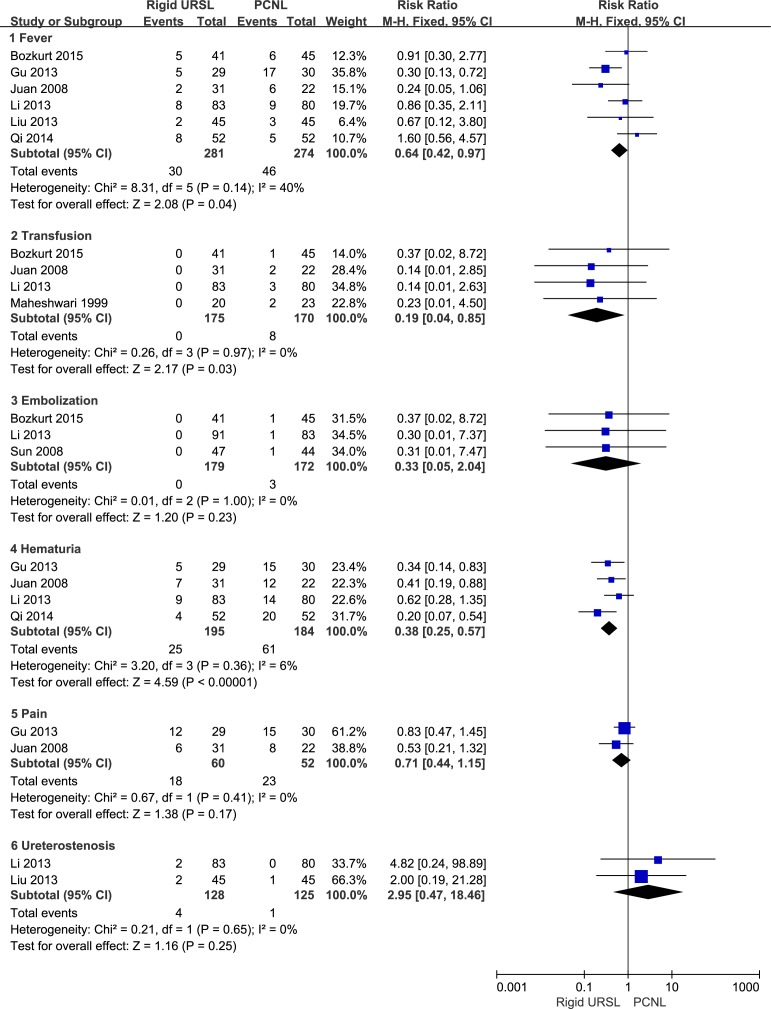
Forest plots of fever, transfusion, embolization, hematuria, pain, and ureterostenosis.

There was no significant difference between rigid URSL and PCNL when it came to pain (RR, 0.71; 95%CI, 0.44 to 1.15; p = 0.17; Fig 6) [14,20], embolization (RR, 0.33; 95%CI, 0.05 to 2.04; p = 0.23; Fig 6) [15,17,19] and ureterostenosis (RR, 2.95; 95%CI, 0.47 to 18.46; p = 0.25; Fig 6) [16,19]. Compared to patients underwent PCNL, those underwent rigid URSL were associated with a lower risk of fever (RR, 0.64; 95%CI, 0.42 to 0.97; p = 0.04; Fig 6) [12,14,16,17,19,20], transfusion (RR, 0.19; 95%CI, 0.04 to 0.85; p = 0.03; Fig 6) [17,19–21] and hematuria (RR, 0.38; 95%CI, 0.25 to 0.57; p < 0.0001; Fig 6) [12,14,19,20].

### Sensitivity analysis

Table 2 showed the results of sensitivity analysis. When only the RCTs [12–16] were included, most of the outcomes including hospital stay, 3^rd^-day stone free rate, 1^st^-month stone free rate, and the incidence of conversion to other surgical procedures, migration, ureteral perforation and postoperative hematuria were stable. However, significant difference in operation time and fever between the two groups could not be observed again. Despite this, the tendency remained the same. When studies [12,14,17,19] mentioning flexible URSL as intraoperative auxiliary procedure were excluded, there came similar results and significant difference in transfusion was no longer detectable.

**Table 2:** Results of sensitivity analysis.

		Sample size	Test for		Test for			
Items	Studies	URSL/ PCNL	Heterogeneity		overall effect		RR / WMD	Favor
			I^2^	P*	Z	P*	95% CI	
**Analysis of RCTs**								
Operation time(min)	[12–16]	220/220	99%	<0.001	1.02	0.31**	-18.08[-52.96,16.80]	**-**
Hospital stay(d)	[12–16]	220/220	92%	<0.001	8.02	<0.001	-3.14 [-3.91, -2.37]	URSL
3-day SFR	[12,13,15,16]	191/190	51%	0.11	4.74	<0.001	0.76 [0.68, 0.85]	PCNL
1-month SFR	[13–16]	139/160	0%	0.93	3.55	0.0004	0.87 [0.81, 0.94]	PCNL
Conversion	[12,14,15]	128/126	0%	0.83	2.09	0.04	4.92 [1.11, 21.86]	PCNL
Migration	[12,14]	81/82	0%	0.71	3.39	0.007	30.40 [4.23, 218.62]	PCNL
Ureteral perforation	[12,14–16]	170/170	0%	0.99	1.58	0.11	3.51 [0.74, 16.65]	PCNL
Fever	[12,14,16]	126/127	65%	0.06	1.67	0.09**	0.61 [0.34, 1.09]	**-**
Hematuria	[12,14]	81/82	0%	0.42	3.98	<0.001	0.26 [0.14, 0.51]	URSL
**Analysis of studies without using flexible ureteroscope**								
Operation time(min)	[13,15,16,18,20,21]	212/213	97%	<0.001	2.76	0.006	-29.28 [-50.05, -8.50]	URSL
Hospital stay(d)	[13,15,16,18,20]	192/190	97%	<0.001	4.67	<0.001	-2.77 [-3.93, -1.61]	URSL
3-day SFR	[13,15,16,21]	159/161	66%	0.03	4.68	<0.001	0.72 [0.62, 0.82]	PCNL
1-month SFR	[13,15,16,18,20]	192/190	24%	0.26	4.83	<0.001	0.82 [0.75, 0.89]	PCNL
Conversion	[15,18,20,21]	120/119	0%	0.77	2.37	0.02	4.81 [1.31, 17.69]	PCNL
Migration	[18,20]	53/52	0%	0.8	2.73	0.006	15.60 [2.18, 111.80]	PCNL
Ureteral perforation	[15,16,21]	109/111	0%	0.94	1.49	0.14	3.84 [0.65, 22.60]	PCNL
Fever	[16,21]	76/67	0%	0.38	1.79	0.07***	0.37 [0.12, 1.10]	**-**
Transfusion	[20,21]	51/45	0%	0.83	1.6	0.11***	0.18 [0.02, 1.48]	**-**

URSL: (rigid) ureteroscopic lithotripsy PCNL: percutaneous nephrolithotomy SFR: stone-free rate.

RR: relative risk WMD: weighted mean difference CI: confidence interval.

* P<0.05 was considered statistically significant

** Originally significant before nRCTs were excluded.

***Originally significant before studies using flexible ureteroscope were excluded.

## Discussion

Large proximal ureteral stones can cause urinary obstruction and some of them are impacted, which prevents the passage of a guidewire or ureteral catheter and enhances the surgical challenge for urologists [27]. Progressive back pressure on the kidney caused by long term obstruction may ultimately lead to significant cortical atrophy and impairment of renal function. Moreover, secondary nephropyosis may be life-threatening [28]. The development of more minimally invasive techniques for treating proximal ureteral stones, such as URSL and PCNL, has largely replaced open surgery.

URSL was first recorded in 1912, and then rapid development of smaller rigid ureteroscopes, reliable laser technology, digital imaging system and flexible instruments has further expanded the indications for its use [29]. Since 1976 Fernström and Johansson firstly introduced PCNL into routine clinical practice, it has been recommended as the first-line treatment of large (>2cm), multiple and inferior calyx renal stones [4]. Although progressive advances in percutaneous approach have been achieved with the advent of mini-, ultramini- and micro-PCNL, PCNL is still associated with a considerable overall complication rate, including perioperative bleeding, fever or sepsis, adjacent organ injury and so on [30,31].

This meta-analysis is the first systematic review comparing the outcomes of rigid URSL and PCNL in treating large proximal ureteral stones. Ten studies (837 patients) were included. No significant difference was observed between the two groups in terms of baseline characteristics, which promised reasonable comparisons.

A higher risk of conversion to another surgical procedures, migration and ureteral perforation was observed in URSL. Upward migration of ureteral stones or big fragments is the main reason for failure in rigid URSL and the reported incidence ranges from 28% to 60% [32], which is generally caused by the increment of irrigation fluid pressure to gain a clear vision and the back-pressure effect of lithotripsy equipment. Although some studies used an anti-retropulsion device such as retrieval basket during rigid URSL, the large impacted stones did not allow additional space for passing the wire of the device and the sever dilation of upper urinary tract sometimes seemed larger than the device [12,14,17]. Moreover, the edematous and inflammatory mucosa or fibroepithelial polyp may result in impede visualization of the stones, which makes it difficult to perform the lithotripsy [33,34]. Sometimes a tortuous ureter or unusual angulation of the ureter also makes it difficult to reach the stones. In these cases, urologists would choose to make conversion to PCNL, laparoscopic or open ureterolithotomy. Ureteral perforation is a serious complication during rigid URSL but most of these perforations are minor and can be managed by ureteral stents [35]. Some severe cases may need conversion to an open ureterolithotomy for further ureteral repair. PCNL is superior in avoiding migration because it is performed via an antegrade tract, which allows it acting as an effective anti-reputation device.

The 3rd-day stone-free rate of PCNL was found to be higher than that of rigid URSL. Upward migrations of ureteral stones or big fragments often lead to incomplete rigid URSL. If a migration happened, an auxiliary procedure such as flexible URSL or SWL would often be needed. Flexible URSL was performed in the same session while SWL was always performed 1-week latter or after the first follow-up. Although flexible ureteroscopy improved the efficiency of URSL, it should be indicated that flexible ureteroscope is still not available in many hospitals in developing countries due to high cost for the equipment. We didn’t conduct a subgroup analysis because there was only one study in this part performing flexible URSL as an auxiliary procedure. Postoperative SWL as an auxiliary procedure was found in five studies [12,14,16,18,21], both the overall and “With postoperative auxiliary SWL” subgroup 1st-month stone-free rate were found lower in rigid URSL, let alone the “Without postoperative auxiliary SWL” subgroup. There might be several superiorities ensure the high efficiency in stone clearance for PCNL. Since most patients with large impacted proximal stones develop hydronephrosis, allowing easier and safer puncture and more space for the nephroscope. Another advantage of PCNL is that an associated renal stones can be removed simultaneously. What’s more, if access to stones which locate below the upper border of the fourth lumbar vertebra is difficult for nephroscope, percutaneous antegrade ureteroscopy through the nephroscope sheath will be a good alternative modality because the rigid ureteroscope can reach the entire upper ureter [36].

We found that it took less time to perform rigid URSL than PCNL. It should be indicated that most studies didn’t state the definition of operation time clearly and each study might calculate the operation time in different criteria, which was also the most important reason for high heterogeneity. Moreover, operation time mainly depends on patient characteristics and surgeon’s experience. The shortest and longest operation time for rigid URSL in included studies was 30.6±7.8min [15] and 92.0±32.5min [17] respectively, while 38.5±8.2min [12] and 115.4±49.5min [20] for PCNL, which showed a great difference.

Rigid URSL yielded significantly shorter hospitalization duration than PCNL. Currently, patients undergoing surgery expect to return to work and to daily activities soon. However, more invasive technique requires more time to make sure that no sever postoperative complications will happen. So after PCNL, patients are often required to lie in bed and limit their activities for several days to reduce the risk of bleeding.

In addition, PCNL was associated with higher risk of transfusion and hematuria than rigid URSL. This result is consistent with the fact that bleeding is generally common in PCNL, which may require transfusion. Actually, a systematic review has reported that the overall transfusion rate is about 7%, indicating the rare requirement of transfusion for PCNL [10]. If conservative measures fail, 0–1.5% cases require selective embolization [11]. Our meta-analysis showed no statistically significant difference of embolization between two groups and ensured the safety of PCNL.

Overall, we found no statistically significant difference in pain, despite the fact that PCNL is more invasive. Although ureteral perforation was more common in rigid URSL according our analysis, most of these perforations were minor and could be managed by ureteral stents. Thus it might be the reason for no statistically significant difference of ureterostenosis. However, only two studies [16,19] referred to ureterostenosis and the follow-up time was not all the same, so more studies should be conducted to prove the reality of this outcome.

There are several limitations in our meta-analysis. Firstly, we defined “large” as stones with a size > 10mm according to EAU guideline [4] but eight studies [12–14,16,17,19–21] in our analysis defined “large” as stones > 15mm. Although the results were the same when we only analyzed the eight studies (data not shown), more studies completely according with our definition were expected to confirm the stability of our analysis. Secondly, the sample size of each study was small and only 837 patients were included. After that, there were only five RCTs in this analysis. Thirdly, although AUA guideline has recommended that flexible ureteroscope should be available when performing URSL for proximal ureteral stones [1], we only focused on rigid URSL in this analysis, which may not represent the latest tendency for ureteroscopic lithotripsy. Fourthly, there existed significant heterogeneities when assessing continuous data such as operation time and hospital stay. Lastly, the funnel plot of operation time, which included all studies, indicated that there might be a publication bias for the included studies.

## Conclusion

In conclusion, our meta-analysis shows that both PCNL and rigid URSL are safe for patients with large proximal ureteral stones, despite the fact that PCNL is associated with a higher risk of transfusion and rigid URSL gets more ureteral perforation. PCNL seems more likely to be successful and is also superior to URSL in stone clearance, which is a very important index to evaluate the efficiency of a surgical technique. More clinical trials in the future need to be conducted to confirm the outcomes of our meta-analysis.

## Supporting information

S1 File PRISMA 2009 checklist. (DOC)
